# Establishment of muscle mass-based indications for the cystatin C test in renal function evaluation

**DOI:** 10.3389/fmed.2022.1021936

**Published:** 2022-11-30

**Authors:** Jisook Yim, Nak-Hoon Son, Kyoung Min Kim, Dukyong Yoon, Yonggeun Cho, Taeyoung Kyong, Ja-Young Moon, Tae Im Yi, Sang-Guk Lee, Yongjung Park, Jung Joo Lee, Kyung-Ah Kim, Jung Eun Lee, Jeong-Ho Kim

**Affiliations:** ^1^Department of Laboratory Medicine, Yonsei University College of Medicine, Seoul, South Korea; ^2^Department of Laboratory Medicine, College of Medicine, The Catholic University of Korea, Seoul, South Korea; ^3^Department of Statistics, Keimyung University, Daegu, South Korea; ^4^Division of Endocrinology, Department of Internal Medicine, Yongin Severance Hospital, Yonsei University College of Medicine, Yongin, South Korea; ^5^Department of Biomedical Systems Informatics, Yongin Severance Hospital, Yonsei University College of Medicine, Yongin, South Korea; ^6^Department of Laboratory Medicine, Hallym University Sacred Heart Hospital, Anyang, South Korea; ^7^Department of Hospital Medicine, Yongin Severance Hospital, Yonsei University College of Medicine, Yongin, South Korea; ^8^Department of Rehabilitation Medicine, Yongin Severance Hospital, Yonsei University College of Medicine, Yongin, South Korea; ^9^Department of Nutrition, Yongin Severance Hospital, Yonsei University College of Medicine, Yongin, South Korea; ^10^Department of Laboratory Medicine, Yongin Severance Hospital, Yonsei University College of Medicine, Yongin, South Korea; ^11^Division of Nephrology, Department of Internal Medicine, Yongin Severance Hospital, Yonsei University College of Medicine, Yongin, South Korea

**Keywords:** bioelectrical impedance analysis (BIA), creatinine (Cr), cystatin C (CysC), estimated glomerular filtration rate (eGFR), muscle mass, kidney function tests, calf circumference

## Abstract

**Background:**

We aimed to suggest muscle mass-based criteria for using of the cystatin C test for the accurate estimated glomerular filtration rate (eGFR).

**Materials and methods:**

We recruited 138 Korean subjects and evaluated eGFRcr (derived from Chronic Kidney Disease Epidemiology Collaboration (CKD-EPI) based on creatinine) was compared to eGFRcys based on cystatin C as the reference value. The skeletal muscle mass index (SMI) by bioelectrical impedance analysis (BIA) was used as representative of muscle mass. Calf circumference (CC) was also evaluated. We defined the patients by eGFRcr as those with values of eGFRcr ≥ 60 mL/min/1.73 m^2^ but eGFRcys < 60 mL/min/1.73 m^2^ as the detection of hidden renal impairment (DHRI). Cut-off values were determined based on muscle mass for the cases of DHRI suggesting the criteria of cystatin C test in renal function evaluation.

**Results:**

We confirmed significant negative correlation between %difference of eGFRcr from eGFRcys and SMI (*r*, −0.592 for male, −0.484 for female) or CC (*r*, −0.646 for male, −0.351 for female). SMI of 7.3 kg/m^2^ for males and 5.7 kg/m^2^ for females were suggested to be significant cutoffs for indication of cystatin C test. We also suggested CC would be valuable for cystatin C indication.

**Conclusion:**

We suggested the muscle mass-based objective criteria relating to SMI and CC that would indicate the use of cystatin C to evaluate renal function test in sarcopenic cases. Our results highlight the importance of muscle mass-based selection of renal function.

## Introduction

Accurate prediction of renal function is important in diagnosing and treating of renal disease, drug dosage adjustment, and contrast agent use (1). Although serum creatinine is the most commonly used marker of renal function, its interpretation is hampered by the variation of muscle mass, and dietary protein intake (2, 3). Creatinine levels could stay within the reference interval despite significant kidney damage in patients with low muscle mass. Frailty, sarcopenia, and malnutrition often occur concomitantly in hospitalized older adults (4). One week of bed rest was reported to reduce skeletal muscle mass substantially; as such, inpatients are at a higher risk of sarcopenia (5). Consequently, serum creatinine is not a good indicator when analyzing the elderly or patients who are expected to have a reduced muscle mass (6, 7).

Unlike that of serum creatinine, the rate of production of cystatin C is not related to muscle mass (7). The independence of cystatin C values from muscle mass is an important advantage for the early detection of kidney damage (8, 9). In many studies of adults and children, cystatin C-based estimated glomerular filtration rate (eGFR) predicted GFR more accurately than did serum creatinine (9–11).

Despite the known advantages of cystatin C, it could also be affected by other factors, such as chronic inflammation, obesity, diabetes, smoking, and thyroid dysfunction, among others (11). Cystatin C test is more expensive than the creatinine test, its standardization is in progress and there are unresolved problems relating to its use, such as uncertainty about insurance coverage of cystatin C and creatinine test simultaneously.

The elderly and inpatients would continue to be the main target of health care. Because the elderly and patients with severe chronic disease requiring long-term hospitalization would have reduced muscle mass, serum creatinine may underestimate the extent of renal dysfunction in this population. This would lead to under-recognition of renal impairment and thus delayed or suboptimal care.

Although it is widely known that creatinine is affected by muscle mass, no studies have provided criteria for using or not using the creatinine test in the evaluation of kidney function. The aim of this study is to determine the objective criteria of cystatin C indication using muscle mass-based parameters for the desirable estimation of GFR.

## Materials and methods

### Subjects

In this study, Korean inpatients and health-check subjects over the age of 40 admitted to Yongin Severance Hospital, a 500-bed capacity secondary care hospital, were recruited for cross-sectional analysis from July 2021 to November 2021. To avoid interference with cystatin C levels, subjects with chronic inflammation (C-reactive protein, CRP > 8 mg/dL), diabetes, obesity (Body Mass Index, BMI ≥ 30 kg/m^2^), thyroid dysfunction, and steroid use (glucocorticoids) were excluded. In addition, to exclude factors that possibly interfere with bioelectrical impedance analysis (BIA), patients with an implanted pacemaker and patients with amputation, ascites, edema, and skin damage to the wrist or ankle were excluded. Finally, 138 adults (male, 57; female, 81) were eligible for enrollment in this study. All enrolled patients were evaluated for BIA (BWA2.0, InBody, Seoul, Korea), anthropometric measurements, serum creatinine, and cystatin C levels. We obtained written informed consent from all participants, and this study was reviewed and approved by the institutional review board of the Yongin Severance Hospital, Yongin-si, Korea (IRB No. 9-2021-0095).

### Measurement and assessment

#### Creatinine and cystatin C

Creatinine and cystatin C were measured in serum samples. Creatinine was measured using the enzymatic method (Roche Creatinine Plus ver.2 assay), which is standardized against the Isotope Dilution-Mass Spectrometry method. Cystatin C was measured using the immunoturbidimetric method (Tina-quant Cystatin C Gen. 2, Roche), which is standardized and traceable against ERM-DA471/IFCC reference material. Both tests were measured using the Roche cobas 8000 c 702 (Roche Diagnostics, Mannheim, Germany).

#### Anthropometric analysis

Anthropometric analysis of mid-arm circumference (MAC), mid-arm muscle circumference (MAMC), and calf circumference (CC) were performed on inpatients (*n* = 66). MAMC was calculated using the following formula: MAMC (cm) = MAC (cm) − 0.314 × triceps skinfold thickness (mm). MAC and CC were measured to the nearest 0.1 cm with a non-elastic tape measure. Triceps skinfold thickness was the average of two measurements taken by the same researcher using a Dynatron skinfold caliper (Dynatronics Corporation, Salt Lake City, UT, USA) on the mid arm area. CC was measured twice, and the average was calculated. The subjects were requested his/her knee raise and bent at right angle in supine position (12) and we measured CC perpendicular to the leg bone at the position of the midpoint between the lateral epicondyle of the distal femur and the prominent point of the fibula lateral malleolus bone.

#### Bioelectrical impedance analysis

We used BWA2.0 (InBody, Seoul, Korea) as a BIA device. BWA2.0 is a multi-frequency BIA device that can measure on supine position. It uses eight different frequencies (1 kHz, 5 kHz, 50 kHz, 250 kHz, 500 kHz, 1 MHz, 2 MHz, and 3 MHz) (13). The subjects were asked to hold their limbs slightly away from their bodies, and measurements were performed according to the manufacturer’s instructions.

#### Formulas and definitions

Two kinds of eGFR, eGFRcr (derived from CKD-EPI_2009 based on creatinine measurement) (14) and eGFRcys (derived from CKD-EPI_2012 based on Cystatin C measurement) (15) were compared. Discrepancies between creatinine and cystatin C-based GFRs might indicate errors of eGFRcr due to low muscle mass. A discordance between eGFRcr and eGFRcys was calculated as eGFR %difference, which was defined as follows: (eGFRcr/eGFRcys − 1) × 100 (%). We defined the patients with the detection of hidden renal impairment (DHRI) by eGFRcr as those with values of eGFRcr ≥ 60 mL/min/1.73 m^2^ and eGFRcys < 60 mL/min/1.73 m^2^. The scenario behind DHRI is when creatinine-based eGFR is within the reference interval due to insufficient muscle mass, while cystatin C-based eGFR shows renal impairment. We also derived cut-off values using DHRI to determine which subjects should undergo cystatin C testing rather than creatinine testing for renal function assessment, based on muscle mass. Appendicular lean muscle mass (ALM) was the sum of muscle mass for four limbs. Skeletal muscle index (SMI) was calculated as ALM divided by height squared (16).

#### Statistical analyses

The Pearson correlation coefficient (*r*) was used to determine the correlation between parameters according to the distribution normality. For continuous variables, analysis was performed using an independent two-sample *t*-test when the analysis was to be divided into two groups. The association of each parameter, such as age, sex, and SMI, with creatinine level, was determined *via* logistic regression for DHRI. The level of significance was defined as a *P*-value < 0.05. Receiver operating characteristic (ROC) curves of SMI and CC were constructed to obtain the optimal cut-off value for sensitive detection of DHRI, which showed fixed 100% sensitivity and best specificity. The significant difference of area under receiver operating characteristic (AUROC) compared to random chance or other AUROC was tested by Delong’s test. Through multivariate linear regression analysis, we confirmed the model fit of the prediction of adjusted SMI and sex and the actual serum creatinine levels (Supplementary Figure 1). Statistical analysis was performed with Analyse-it version 5.92 for Microsoft Excel (Analyse-it Software Ltd., Leeds, UK).

## Results

### Study population and baseline characteristics

A total of 138 inpatients and health-check examinees were enrolled in this cross-sectional analysis. The basic characteristics of the study population were classified according to sex (57 males and 81 females) and purpose of visit (inpatients 66, health-check 72), and the baseline characteristics are presented in Table 1. eGFRcr and eGFRcys values did not show significant differences between sex groups. Conversely, the comparison between the inpatient and the health-check group were shown significant differences in age, eGFRcys, and SMI, while eGFRcr did not.

**TABLE 1:** Baseline characteristics of the enrolled study population.

Characteristics	Enrolled inpatients and health-check examinees (*n* = 138)					
	Sex			Visiting purpose		
	Male	Female	*P*-value**	Inpatients	Health-check	*P*-value**
Number of subjects (n)	57	81		66	72	
Age (year)	66.4 (13.6)*	67.1 (12.1)	0.7400	73.5 (10.4)	60.8 (11.6)	<0.0001
Age range (year)	40∼93	41∼95	–	41∼95	40∼83	–
Creatinine (mg/dL)	0.852 (0.154)	0.638 (0.123)	<0.0001	0.692 (0.173)	0.759 (0.166)	0.0215
Cystatin C (mg/L)	0.984 (0.181)	0.905 (0.156)	0.0073	0.887 (0.120)	0.993 (0.199)	0.0002
^†^eGFRcr (mL/min/1.73 m^2^)	89.2 (11.8)	90.8 (12.7)	0.4511	88.1 (12.0)	92.0 (12.4)	0.0651
^‡^eGFRcys (mL/min/1.73 m^2^)	80.8 (16.7)	82.2 (16.6)	0.6296	74.6 (16.8)	88.0 (13.6)	<0.0001
^‡^eGFRcr + cys (mL/min/1.73 m^2^)	85.5 (13.3)	88.3 (15.0)	0.2486	91.4 (12.8)	82.5 (14.6)	0.0002
§Calf circumference (cm)	31.69 (3.48)	29.85 (3.09)	0.0279	30.60 (3.35)	–	–
§Mid-arm muscle circumference (cm)	20.76 (3.24)	17.38 (1.97)	<0.0001	18.77 (3.04)	–	–
SMI by BIA (kg/m^2^)	7.40 (1.11)	5.86 (0.73)	<0.0001	6.08 (1.17)	6.88 (1.06)	<0.0001

*Mean (standard deviation), all such values.

**Independent two-sample *t*-test.

^†^Estimated by the Chronic Kidney Disease Epidemiology Collaboration (CKD-EPI) creatinine equation, 2009 version (14).

^‡^Estimated by the CKD-EPI cystatin C equation, 2012 version, and CKD-EPI creatinine + cystatin C equation, 2012 version (15).

§Calf circumferences and mid-arm muscle circumference were measured only for inpatients (*n* = 66). SMI, skeletal muscle mass index adjusted by height squared; eGFR, estimated glomerular filtration rate; BIA, bioelectrical impedance analysis; CC, calf circumference.

### Correlation between skeletal muscle mass index and creatinine

We obtained the following equation and fit model for serum creatinine (Supplementary Figure 1). SMI and sex could explain 44.1% of the serum creatinine levels by multiple regression analysis (*r*^2^ adjusted = 0.441).

Serum Creatinine = 0.3329 + 0.0521 SMI + 0.1337 Sex (*P*-value < 0.0001; SMI as kg/m^2^; for sex, males = 1, females = 0).

### Correlation among skeletal muscle mass index, mid-arm muscle circumference, calf circumference, and estimated glomerular filtration rate %difference

**FIGURE 1:**
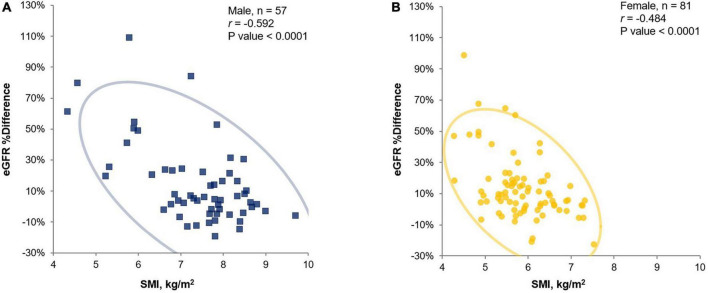
Pearson’s correlation analysis for eGFR %difference and SMI (by BIA InBody BWA2.0 model) in male **(A)** and female **(B)** cross-sectional study participants (inpatients and health-check examinee). eGFR, estimated glomerular filtration rate; SMI, skeletal muscle mass index adjusted by height squared; BIA, bioelectrical impedance analysis.

There were significant negative correlations between SMI and eGFR %differences (*r* = −0.592 [95% CI −0.739 to −0.392] for males and *r* = −0.484 [95% CI −0.635 to −0.297] for females; *P*-value < 0.0001 for both sexes) (Figure 1). We analyzed the correlation between SMI or eGFR %difference and other related parameters, such as MAMC and CC. Significant positive correlation with SMI was shown for CC (*r* = 0.902 [95% CI, 0.795 to 0.955], *P*-value < 0.0001 for males; *r* = 0.687 [95% CI, 0.475 to 0.824], *P*-value < 0.0001 for females) (Supplementary Figure 2). CC and eGFR %differences showed significant negative correlation (*r* = −0.646 [95% CI −0.824 to −0.353], *P*-value = 0.0003 for males; *r* = −0.351 [95% CI −0.600 to −0.040] for females, *P*-value = 0.0285) (Supplementary Figure 3). We could also find significant correlation between SMI and other parameters, such as MAMC (*r* = 0.608 [95% CI, 0.297 to 0.803]; *P*-value = 0.0008 for males and *r* = 0.412 [95% CI, 0.111 to 0.644]; *P*-value = 0.0092 for females). Another significant negative correlation was shown between eGFR %difference and MAMC only for males (*n* = 27; *r* = −0.421 [95% CI −0.691 to 0.049], *P*-value = 0.0286) but not for females (*n* = 39; *r* = −0.263 [95% CI −0.534 to 0.057], *P*-value = 0.1054).

### Comparison between the inpatient and health-check groups

**FIGURE 2:**
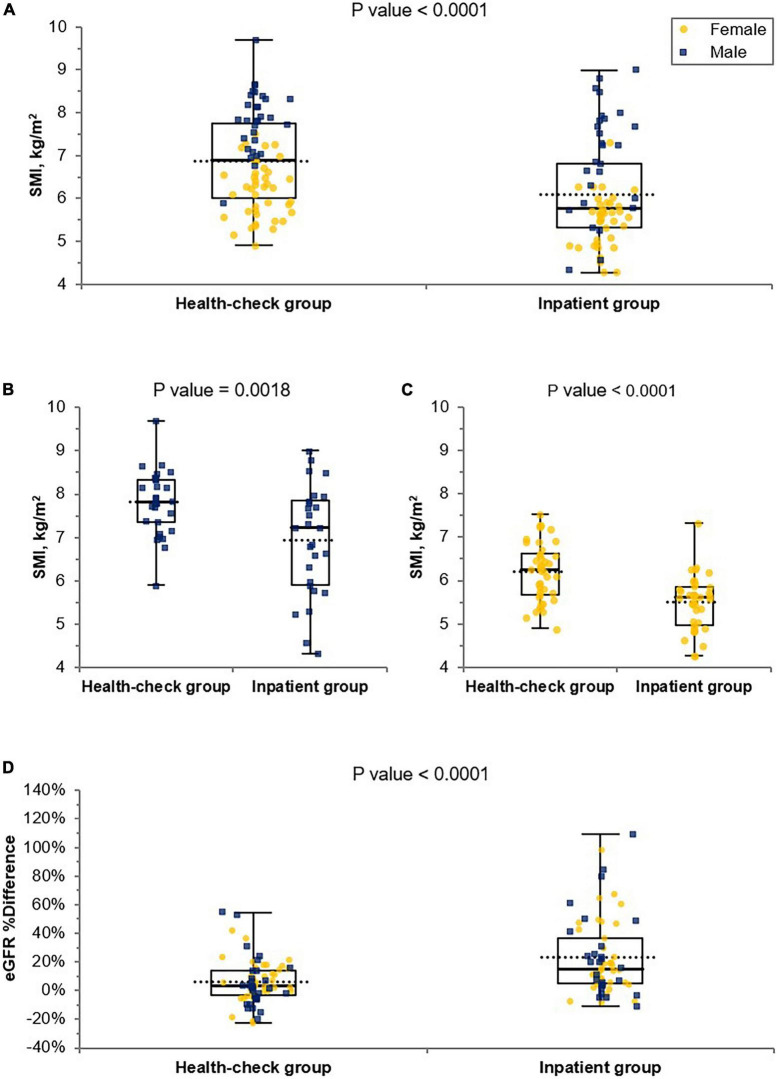
Comparison of SMI and eGFR %differences in the inpatient and health-check groups. A significance decrease in SMI in both sexes **(A)**, in male **(B)** and female **(C)** was confirmed in the inpatient group compared to that in the health-check group by Student’s *t*-test. A significant increase for eGFR %difference was confirmed in the inpatient group compared to that in the health-check group by Student’s *t*-test **(D)**. The blue square dots represent each male subject, and the yellow circles represent each female subject. eGFR, estimated glomerular filtration rate; eGFR %difference, % difference between eGFRcr and eGFRcys; SMI, skeletal muscle mass index adjusted by height squared.

Upon comparison between the inpatient and health-check groups showed in Figure 2, a significant decrease in SMI in both sexes (A), in males (B), and females (C) was confirmed in the inpatient group compared to that in the health-check group. A significant increase for eGFR %difference was confirmed in the inpatient group compared to that in the health-check group (D).

### Establishment of cut-off values to guide the cystatin C test

**FIGURE 3:**
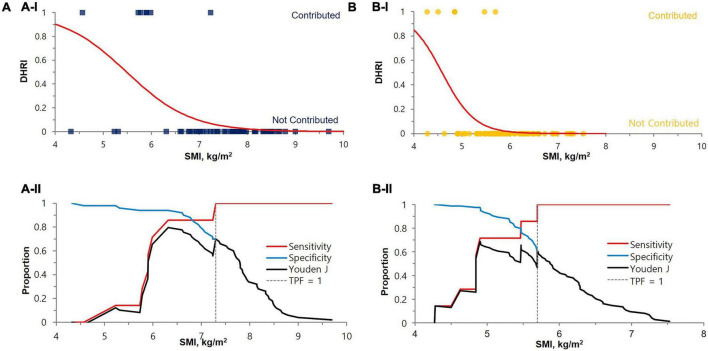
Cut-off value determination by SMI for the cystatin C test indication for renal function test. The cut-off values were 7.3 kg/m^2^ for males (*P-*value < 0.0001) **(A)** and 5.7 kg/m^2^ for females (*P*-value < 0.0001) **(B)**, either by (I) Logistic regression or (II) Decision threshold. In the upper **(A-I)** and **(B-I)** logistic regression graphs, the contributed case for DHRI is denoted as 1 and non-contributed case is denoted as 0. The lower **(A-II)** and **(B-II)** are decision threshold graphs showing fixed 100% sensitivity and best specificity (70% for males and 61% for females). The blue square dots represent each male subject, and the yellow circles represent each female subject. eGFR, estimated glomerular filtration rate; SMI, skeletal muscle mass index adjusted by height squared; DHRI, detection of hidden renal impairment case defined as eGFRcr ≥ 60 mL/min/1.73m^2^ and eGFRcys < 60 mL/min/1.73 m^2^; TPF, true positive fraction.

**FIGURE 4:**
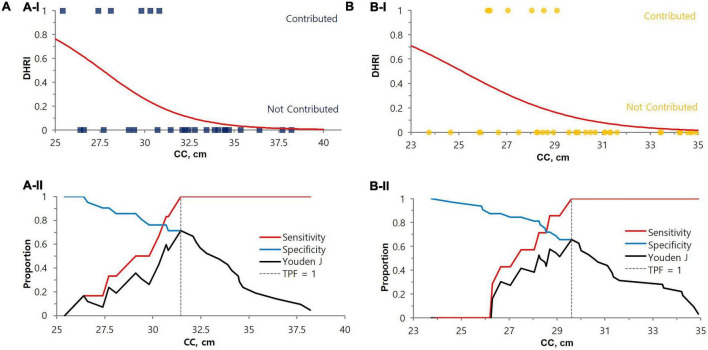
Cut-off value determination by CC for the cystatin C test indication for renal function evaluation. The cut-off values for DHRI were 31.5 cm for males (*P*-value = 0.0081) **(A)** and 29.6 cm for females (*P*-value = 0.0111) **(B)**, either by (I) Logistic regression or (II) Decision threshold. In the upper **(A-I)** and **(B-I)** logistic regression graphs, the contributed case for DHRI is denoted as 1 and non-contributed case is denoted as 0. The lower **(A-II)** and **(B-II)** are decision threshold graph s showing fixed 100% sensitivity and best specificity (71% for males and 66% for females). The blue square dots represent each male subject, and the yellow circles represent each female subject. eGFR, estimated glomerular filtration rate; DHRI, detection of hidden renal impairment case defined as eGFRcr ≥ 60 mL/min/1.73 m^2^ and eGFRcys < 60 mL/min/1.73 m^2^; CC, calf circumference; TPF, true positive fraction.

**FIGURE 5:**
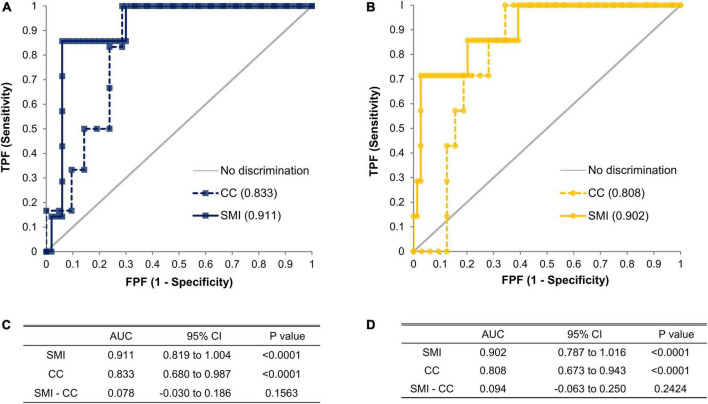
ROC curves of SMI and CC in males **(A)** and females **(B)**. The significant difference of area under receiver operating characteristic (AUROC) compared to random chance or other AUROC was tested by Delong’s test in terms of SMI (*n* = 57 for male, *n* = 81 for female) and CC (*n* = 27 for male, *n* = 39 for female). AUC with its 95% confidence intervals and *p*-values of AUCs of SMI and CC, and its difference in males **(C)** and females **(D)**. ROC, receiver operating characteristic curve; AUROC, area under receiver operating characteristic; AUC, area under the curve; SMI, skeletal muscle mass index adjusted by height squared; CC, calf circumference; CI, confidence interval.

We performed logistic regression analysis accounting for SMI and DHRI to determine cutoff values that would indicate a recommendation for cystatin C rather than creatinine testing for renal function evaluation (Figure 3). We determined the cutoff values for having a cystatin C test rather than a creatinine test for renal function evaluation to be a SMI value of 7.3 kg/m^2^ for males and 5.7 kg/m^2^ for females. We determined a CC value of 31.5 cm for males and 29.6 cm for females as cutoff values indicating a preferential cystatin C test (Figure 4). We could not find a significant threshold for MAMC for cystatin C test indication (data not shown). Flowchart for the selection of appropriate renal function test according to muscle mass is proposed in Supplementary Figure 4. Figure 5 shows the ROC curves and *P*-values of AUCs of SMI and CC, showing that the AUC of CC was 0.833 for males and 0.808 for females. AUC of SMI was 0.911 (95% CI = 0.819 to 1.004, *P*-value < 0.0001) for males and 0.902 (95% CI = 0.787 to 1.016, *P*-value < 0.0001) for females. There was no significant difference between the AUCs of SMI and CC in both sexes (Figure 5D). When our cutoff was applied, there were a total of 14 cases of DHRI among sarcopenia patients (1 check-up subject, 13 hospitalized patients). We calculated the proportion of the cases of that cystatin C is required in 43 to 47% of our study subjects due to low muscle mass based on SMI and CC, respectively.

### Optimal estimated glomerular filtration rate equations according to muscle mass

We compared the results calculated with the eGFRcr formula and those of the formula based on both creatinine and cystatin C Chronic Kidney Disease Epidemiology Collaboration (CKD-EPI) eGFR (eGFRcr + cys), to those of the eGFRcys formula. Patients were categorized into the sarcopenia and non-sarcopenia groups based on the obtained cut-off values (7.3 kg/m^2^ for males and 5.7 kg/m^2^ for females) for SMI in the present study. The percentages falling within ±30, ±20, and ±10% of the eGFRcys results were defined as P30, P20, and P10, respectively (Supplementary Table 1).

## Discussion

Although serum creatinine is widely used as an indicator of GFR, it is not a sensitive indicator of renal function, as the GFR may need to decrease by >50% before serum creatinine is outside the broad reference interval (17). Creatinine is also affected by various interferences such as sex, age, muscle mass, and dietary protein intake, among other factors (2, 3, 18). Among these interfering factors, muscle mass is known to affect creatinine levels markedly (9). Formulas for creatinine-based eGFR take sex, age, and weight into account as surrogates for muscle mass, because direct muscle mass measurement is clinically difficult (19, 20). Nevertheless, as these eGFR formulas still had unsolved fundamental problems relating to creatinine, such as having a wide reference interval for normal levels of creatinine, displaying results with reduced sensitivity, and not taking muscle mass into account, eGFRcr could be within the reference interval in individuals with low muscle mass, even with actually impaired renal function.

Many studies that have examined the effect of creatinine according to muscle mass reported a clinically significant difference between inferred and actual renal function (6, 7, 9, 21). The superiority of cystatin C in renal function evaluation for populations with relatively lower muscle masses, such as the elderly, children, or women, has been agreed upon in many previous studies (6, 7, 9). However, information about measuring muscle mass and the actual criteria to perform the cystatin C test is lacking. Based on these concerns, in our study, objective muscle mass was measured and the effect of muscle mass on creatinine and creatinine-based eGFR was analyzed. In addition, based on the muscle mass, criteria for performing the cystatin C test instead of the creatinine test were derived.

In the comparison between hospitalized patients and health-check subjects, there was no significant difference between eGFRcr values, while eGFRcys showed a significant difference, as shown in Table 1. Likewise, a significantly larger increase in eGFR %difference values in the inpatient group compared to that in the health-check group could be interpreted in a similar context; these results are probably because the former group includes elderly patients and patients with sarcopenia (Figure 2). These results imply that eGFRcr could mislead the cases of impaired renal function with low muscle mass as normal renal function, as reported in previous studies (6, 9), and would be an explanation for why the results of our study are different from those of Swaminathan et al.’s (17) study, which had been conducted on healthy subjects. According to our study results, if renal function is evaluated in subjects whose eGFRcr is not impaired, especially in hospitalized patients, muscle mass evaluation would be necessary to determine the presence of sarcopenia. Furthermore, if there is sarcopenia, it would be preferable to perform a cystatin C test rather than a creatinine test to obtain an appropriate renal function result.

Popular muscle mass assessment tools include body imaging techniques (e.g., MRI, CT, dual-energy X-ray absorptiometry (DXA), and ultrasonography), BIA, anthropometric parameters (e.g., CC and MAMC), and biochemical markers (total or partial body potassium, serum and urinary creatinine, and deuterated creatine dilution method) (22). However, even though other methods such as MRI, CT, and DXA, which have been previously introduced as methods to measure muscle mass, using precise imaging technology, these modalities are expensive, may entail radiation exposure, and require patient transport. These methods are limited in terms of feasibility. Practicality, accuracy, and cost are important factors in choosing any method in clinical practice. BIA is an appropriate method of measuring muscle mass for our purposes. MacDonald et al. (23) mentioned that ALM by BIA provides a clinically obtainable and valid method to predict muscle mass in patients with chronic kidney disease; the improvement of GFR_inulin_ estimation upon using ALM by BIA has also been reported. The correlation between BIA and other muscle mass measurement methods, such as DXA, has been studied extensively in measuring muscle mass (24–27). BIA models that could be viable to patients in the supine position have been developed recently, such as the S10 (InBody, Seoul, Korea) (28); it seems to be appropriate for critically ill patients or inpatients who have difficulty in standing or ambulation (26, 29, 30). In this study, we used the upgraded model (BWA2.0) for S10, which can be used for the patient with a supine position, BWA2.0 model had been recently validated against DXA (13). Furthermore, our study showed that measuring CC would be a good alternative valid method for the assessment of sarcopenia.

We found that SMI or CC can be a significant parameter to indicate the need for testing for cystatin C levels. The sarcopenic cutoffs we obtained (SMI < 7.3 kg/m2 for males, <5.7 kg/m2 for females by BIA), were similar to those reported by the Asian Working Group for Sarcopenia (AWGS) 2019 (16). Low CC (<31.5 cm for males, <29.6 cm for females) would be an alternative parameter to indicate cystatin C preference for possible sarcopenia. The CC cutoffs we obtained were much lower than those of the AWGS 2019 (<34 cm in males and <33 cm in females) (16) or another Korean study (<35 cm in males and <33 cm in females) (31). Suspected reasons for lower CC cutoffs of our results than others are considered as follows: we did not measure the largest girth of the calf, but measured the anatomical midpoint between the lateral epicondyle of the distal femur and the prominent point of the fibula lateral malleolus bone. And also our study included elderly hospitalized patients, and might be lower extremity muscles are more reduced than other muscles in these patients. In our study, we found that a cystatin C test may be required in addition to creatinine levels to detect hidden renal impairment in 43 and 47% of our study subjects due to low muscle mass detected by SMI and CC, respectively. Creatinine tests seemed to be sufficient for the remaining proportions (57, and 53%, respectively) of patients. According to the Kidney Disease: Improving Global Outcomes (KDIGO) guideline for the other cystatin C indications such as borderline eGFRcr between 45 and 74 ml/min/1.73 m^2^ or persons at high risk of CKD, more subjects might require to be tested for cystatin C (11, 32).

When we set eGFRcys as a reference, we found that P30 of eGFRcr was significantly decreased from 93.6% in the non-sarcopenic group to 70.0% in the sarcopenic group as in Supplementary Table 1. If we use the eGFRcr + cys as recommended by Inker et al. (15), P30 showed values above 80% for both the non-sarcopenic and sarcopenic groups, 100 and 90%, respectively. Although it is not appropriate to obtain eGFR by a single marker, namely, creatinine, in the sarcopenic group, creatinine is still applicable as a good single marker in the non-sarcopenic group (Supplementary Table 1).

Our study has several strengths, as follows. Although creatinine and eGFRcr are not suitable for assessing kidney dysfunction in patients with low muscle mass, there have been no objective criteria for when creatinine levels are not valid or cystatin C test is required. To the best of our knowledge, this is the first study to provide an objective muscle mass criterion for testing cystatin C. Considering the missed or delayed diagnosis of renal impairment in the population of patients with low muscle mass, suggested criteria in our study for obtaining cystatin C levels instead of creatinine levels might be helpful. In addition, unlike MRI, CT, and DXA, the methods for muscle mass measurement suggested by this study have practical value in that they could be applied to relevant clinical practice. However, there are several limitations to this cross-sectional study, as follows. Firstly, we could not use exogenous markers that directly determine measured GFR (mGFR). Thus, we were unable to estimate the actual true bias of the eGFRcr values in this study compared to that of the mGFR. However, we tried to detect differences between eGFRcr and eGFRcys due to muscle mass, using eGFRcys as a reference among the subjects, excluding those with factors affecting the measurement of cystatin C. Secondly, we determined muscle mass with multi-frequency BIA or CC rather than DXA, which is currently considered to be a reference method for the evaluation of muscle mass (33). However, some prediction equations have been suggested to rectify the inaccuracy of multi-frequency BIA (24, 26, 27), which generally shows good agreement with DXA (25, 34) and can be used for muscle mass evaluation. Thirdly, various models of multi-frequency BIA from different manufacturers have not been standardized. There have been some reports of slightly different prediction equations according to the type of multi-frequency BIA, but we hypothesize that the differences would be small (29, 30). Fourthly, CC measurement was not fully standardized and was done in a supine position and it may be different from those of sitting or standing position and we measured CC at the midpoint of the lower leg, seemed to be shorter than the largest circumference. Fifth, despite the expected clinical usefulness and feasibility, we could not validate, nor confirm the clinical impact of this study. Sixth, sarcopenic evaluation by BIA is not seemed to be cost-effective just for the cystatin C indication, but BIA could be utilized due to their increasing use in hospitalized patients for sarcopenia, frailty or nutritional assessment. Moreover, CC is very economic and promising method in terms of nearly comparable performance to BIA for sarcopenic evaluation from this study. Finally, we could not enroll a larger population and/or various ethnic groups, and could not obtain enough power to discern clear differences between various parameters.

In this study, the criteria for selecting the cystatin C test or measuring GFR directly (3), rather than the creatinine test were presented in any subjects with apparently reduced muscle mass, especially in the elderly or hospitalized ones, according to the objective muscle mass. Our results highlight the importance of muscle mass-based evaluation of renal function. Further investigations may be necessary for the validation of low muscle mass cutoffs and their clinical impact.

## Data availability statement

The datasets used and/or analyzed during this study are available from the corresponding author upon reasonable request.

## Ethics statement

The studies involving human participants were reviewed and approved by the Institutional Review Board of the Yongin Severance Hospital, Yongin-si, Korea. The patients/participants provided their written informed consent to participate in this study.

## Author contributions

JY, N-HS, TK, TIY, JEL, and J-HK conceived and designed the study. K-AK, J-YM, JJL, and J-HK recruited the subjects and acquired the data. N-HS carried out the statistical consultation. JY, KMK, DY, YC, S-GL, YP, JEL, and J-HK carried out the data interpretation and critical discussion. JY, YP, JEL, and J-HK drafted and revised the manuscript. All authors have read and agreed to the published version of this manuscript.
