# Supplementary material for: Establishment of muscle mass-based indications for the cystatin C test in renal function evaluation
Source: Front Med (Lausanne). 2022 Nov 30;9:1021936. doi: 10.3389/fmed.2022.1021936 (PMC9747759; doi:10.3389/fmed.2022.1021936)
Supplement: Supplementary file 1 [file Data_Sheet_1.docx]

Supplementary Material

## Supplementary Figures


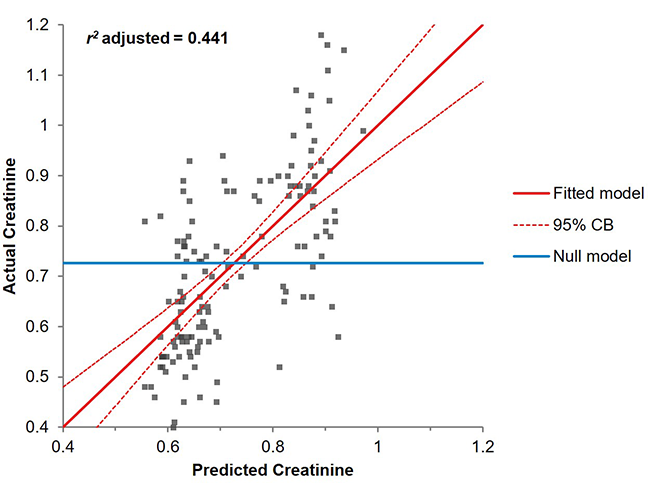


**Figure S1.** The fitted model between actual and predicted serum creatinine values by multiple regression analysis for creatinine, SMI, and sex. The null hypothesis was rejected, with a P value <0.0001 by the F-test.

¶Abbreviations: SMI, skeletal muscle mass index adjusted by height squared; CB, confidence bounds


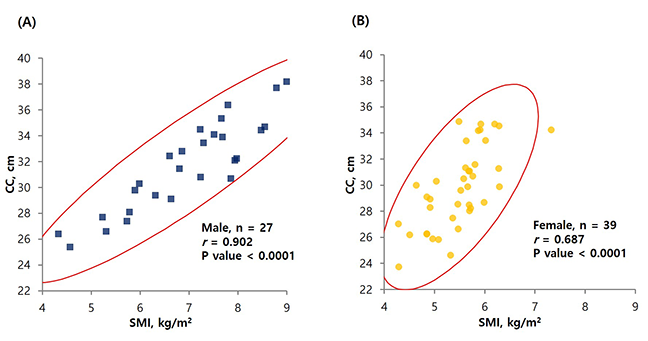


**Figure S2**. Scatter plot for correlation between CC and SMI (A) for males and (B) for females. Pearson’s correlation was used.

¶Abbreviations: CC, calf circumference; SMI, skeletal muscle mass index adjusted by height squared; *r*, coefficient of correlation


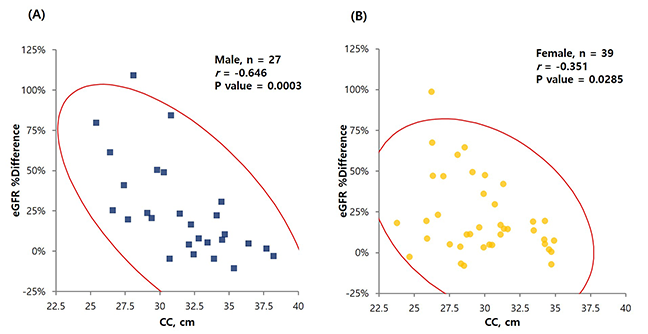


**Figure S3**. Scatter plot for correlation between CC and eGFR %difference (A) for males and (B) for females. Pearson’s correlation was used.

¶Abbreviations: CC, calf circumference; *r*, coefficient of correlation; eGFR, estimated glomerular filtration rate


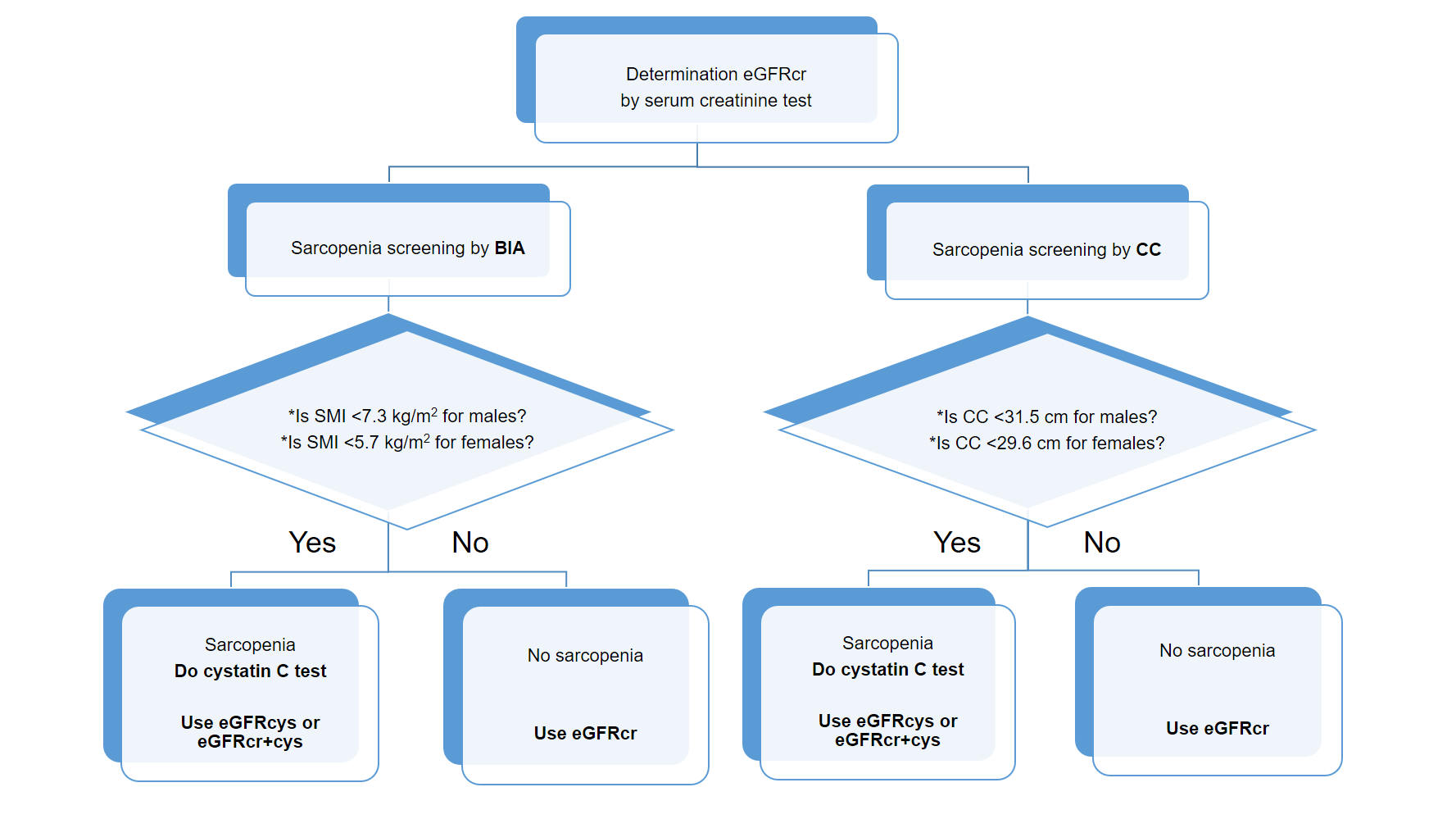
**Figure S4**. Proposed flowchart for selection appropriate renal function test according to muscle mass.

*These cut-offs might not be applicable due to limited study subjects. We did not include the other indications for cystatin C, such as borderline eGFRcr, or persons at high risk of CKD (Reference #11 or #32), in this flowchart.

¶Abbreviations: BIA, bioelectrical impedance analysis using BWA2.0 (InBody, Seoul, Korea); CC, calf circumference. CC was measured with non-elastic tape at the midpoint between the lateral epicondyle of the distal femur and the prominent point of the fibula lateral malleolus bone.

## Supplementary table

**Table S1.** Proportion of P30, P20, and P10 of eGFRcr or eGFRcr+cys based on eGFRcys according to the presence of sarcopenia by SMI measured by BIA.

|  | eGFRcr | | |  | eGFRcr+cys | | |
| --- | --- | --- | --- | --- | --- | --- | --- |
|  | P30 | P20 | P10 |  | P30 | P20 | P10 |
| Non-sarcopenia | 93.60% | 85.90% | 59.00% |  | 100.00% | 97.40% | 82.10% |
| Sarcopenia | 70.00% | 58.30% | 36.70% |  | 90.00% | 73.30% | 41.70% |

^¶^Abbreviations: eGFRcr, creatinine-based CKD-EPI eGFR; eGFRcr+cys, creatinine and cystatin C-based CKD-EPI eGFR, SMI, skeletal muscle mass index adjusted by height squared; eGFR, estimated glomerular filtration rate

†Non-sarcopenia and sarcopenia group classification was based on the obtained cut-off values (7.3 kg/m^2^ for male and 5.7 kg/m^2^ for female) for SMI from present study.

‡The percentages falling within ±30%, ±20% and ±10% to the eGFRcys results were defined as P30, P20, and P10, respectively.
